# Editorial: Structural, Metabolic, and Physiologic MR Imaging to Study Glioblastomas

**DOI:** 10.3389/fneur.2022.887027

**Published:** 2022-03-30

**Authors:** Manoj Kumar, Ravi Prakash Reddy Nanga, Sanjeev Chawla

**Affiliations:** ^1^Department of Neuroimaging and Intervention Radiology, National Institute of Mental Health and Neurosciences, Bangalore, India; ^2^Department of Radiology, Perelman School of Medicine at the University of Pennsylvania, Philadelphia, PA, United States

**Keywords:** glioblastoma, radiomics, metabolic MR imaging, physiologic MR imaging, brain tumor

Glioblastoma (GBM) is the most common and fatal primary malignant brain neoplasm in adults (1). The current standard of care treatment for GBM comprises maximal safe surgical resection followed by concurrent chemoradiation therapy (CCRT) and maintenance chemotherapy with temozolomide. Despite aggressive multimodal treatment the prognosis has remained poor (2). Recently, novel therapeutic approaches such as immunotherapy (3) and electric field therapy (4) have been introduced. Currently, several clinical trials are in progress to evaluate the safety profile and therapeutic efficacy of these new frontiers in fight against this devastating and life-threatening disease.

In the field of neuro-oncology, diagnosis and treatment response evaluation remain highly dependent on neuroimaging methods. While conventional magnetic resonance (MR) imaging sequences provide valuable information about the anatomic details and blood-brain-barrier (BBB) integrity, they lack specificity in characterizing gliomas as these neoplasms are highly heterogeneous both in spatial and temporal dimensions. Continuous developments in metabolic and physiologic MR imaging techniques have provided new insights into understanding underlying tumor biology and tumor microenvironment (5–9). Taken together, these techniques have been utilized to make the correct diagnosis, prognosis, evaluation of treatment response to both established and novel therapeutic regimens, and identification of new molecular targets for fostering the discovery of new treatments. Additionally, an emerging field of “*radiomics*” has the potential to change the ways in which advanced MR imaging techniques can be utilized more efficiently (10). This Research Topic was launched to collect high-quality manuscripts to advance our knowledge on clinical utilities, existing challenges, and limitations of using metabolic and physiologic MR imaging techniques in characterizing GBMs. A total of twelve manuscripts (nine original research and three review articles) were finally accepted for publication under this Research Topic.

## Original Research Articles

The leakage of the contrast agent into the extravascular extracellular space (EES) during the dynamic susceptibility contrast (DSC)-perfusion MRI affects the signals produced in two competing ways. Contrast agent shortens T_1_ values of tissue water within the EES, thereby increasing MR signal results in an underestimation of cerebral blood volume (CBV). Conversely, T_2_^*^-effects caused by changes in susceptibility differences between EES and intravascular compartments reduce the MR signal, which does not recover to baseline during the DSC scan. This effect causes an overestimation of CBV. When the contrast agent extravasation is especially fast (as in malignant gliomas), CBV can be calculated as a negative estimate because the signal increase caused by T_1_ effects is greater than the signal reduction due to T_2_^*^ effects. To address this problem of contrast leakage in gliomas, Arzanforoosh et al. investigated the effect of two known leakage correction algorithms on CBV measurements. The leakage correction algorithms were based on unidirectional contrast agent transport from the intravascular to EES and bidirectional contrast agent transport between these two compartments. The investigators reported that in enhancing gliomas (situations when the BBB is generally disrupted), applying either of these two leakage correction methods decreased CBV measurements.

While exploring the potential associations between molecular features and patterns of contrast enhancement in GBMs, Yang et al. identified endothelial cell-enriched genes from transcriptome data from the GBM patients. The investigators demonstrated that contrast enhancement was associated with distinct vascular molecular imprints which is characterized by up-regulation of proinflammatory genes and de-regulation of BBB-related genes in endothelial cells. Moreover, high contrast enhancement was associated with poor patient prognosis and survival outcomes. Furthermore, enhancing volume/complete tumor volume ratio was significantly higher in the mesenchymal subtype of GBMs.

Huang et al. have suggested that neuroplasticity in patients with insular glioma might play a crucial role in preserving the neurological functions and subsequently improving the post-resection prognosis. The authors correlated gene expression profiles of isocitrate dehydrogenase (IDH), telomerase reverse transcriptase (TERT), and 1p19q codeletions with MRI volumetric data. The authors observed that IDH mutation status was the only genotype that was found to be associated with significant structural compensation in patients with insular glioma. These authors suggested that such findings may help in predicting neurocognitive and functional outcomes in patients with insular glioma undergoing surgical resection.

Adult supratentorial extra ventricular ependymoma (STEE) are rare neoplasms often misdiagnosed as high-grade gliomas (HGG) due to their similar characteristics on conventional neuroimaging. Safai et al. developed a machine learning-based diagnostic model by using quantitative radiomic signatures from multi-model MRI data for distinguishing adult STEE from HGG. The investigators reported that texture-based radiomic features from T_2_-FLAIR images were vital in discriminating STEE from GBM. On the other hand, first-order features from T_2_-weighted images and apparent diffusion coefficient (ADC) maps were consistently ranked higher in differentiating multiple tumor groups.

Against the backdrop of growing concern over patients' radiation exposure from undergoing 2-deoxy-2-[fluorine-18]fluoro-D-glucose (^18^F-FDG)-positron emission tomography (PET) and other inherent shortcomings associated with this technique, Mangalore et al. explored whether high “b” value diffusion-weighted imaging (DWI) and ^18^FDG-PET can provide similar or complementary information in detecting malignant brain lesions. The investigators obtained comparable sensitivity and specificity for DWI and ^18^FDG-PET derived parameters and concluded that DWI could act as a surrogate for ^18^FDG-PET in the diagnosis of brain tumors.

Despite the clinical importance, the accurate distinction between GBMs and solitary metastasis often remains challenging as these entities exhibit similar features on conventional neuroimaging. To address this issue, Zhang et al. designed a clinical trial and developed an integrated radiomics model by incorporating DWI-derived ADC and ^18^FDG-PET derived standardized uptake value (SUV). This integrated model provided significantly better diagnostic performance than the utilization of any single imaging parameter alone.

Using multivariate logistic regression analyses, Wong et al. developed a mathematical model by incorporating multiple clinical features and imaging factors (DWI-derived ADC, contrast-enhancement size, whole-tumor size) in predicting malignant transformation of low-grade gliomas. This model had an accuracy of 84% from the training group and 85% from the validation group.

In a seminal study, Cui et al. investigated the prognostic significance of metabolic alterations from the postoperative peritumoral edematous zone (PEZ) in GBMs. Authors proposed that an elevated choline/N-acetyl aspartate ratio in PEZ can be considered as an independent risk factor for predicting early tumor recurrence. Moreover, this metabolic abnormality was also associated with poor prognosis and adverse clinical outcomes in patients with GBM.

By leveraging the utility of unique brain functional connectivity information obtained from resting-state functional MRI combined with machine learning algorithms, Lamichhane et al. classified GBM patients into short-term and long-term survival groups with high sensitivity and specificity (*precision prognostics*).

## Review Articles

Determining the utility of interval imaging (i.e., imaging at pre-planned time-points to assess tumor status) in brain tumor management remains crucial in neuro-oncology. An expert panel comprising of professionals from data science, health economics, trial management of adult brain tumors, and patient representatives extensively reviewed the current evidence on the use of interval imaging to monitor brain tumors, and summarized their findings in a review article. The investigators concluded that evidence for the value of regular interval imaging is currently lacking. At the same time, the authors indicated that ongoing collaborative efforts might provide some evidence to optimize monitoring imaging biomarkers for the standard of care brain tumor management (Booth et al.).

A review article by Kumar et al. comprehensively showcased the clinical potentials of emerging metabolic imaging techniques such as 3D-echoplanar spectroscopic imaging, 2D-correlation spectroscopy, and chemical exchange saturation transfer imaging in several neuro-oncologic applications. The authors also provided a detailed head-to-head comparison of these three metabolic techniques.

In another review article, Gonçalves et al. provided an overview on the potential utilities of advanced MR imaging techniques for studying pediatric GBMs.

Altogether, the studies published in this special issue have highlighted the importance of using advanced MR imaging and PET imaging techniques in redefining and reshaping our understanding of GBMs. Collectively, these techniques provide crucial information about the tumor microstructure, microvasculature, and metabolism, thus offering opportunities for optimizing clinical care of glioma patients. “*Radiomics*” is a relatively young and evolving field, and has a tremendous potential to provide meaningful biological understandings of imaging features for further improvement in the clinical outcomes and quality of life of these patients. However, the widespread translation of advanced imaging and radiomics methods into the routine clinical workflow has been slow due to some technical challenges. We believe that standardization as well as harmonization of data acquisition and post-processing procedures will strengthen the clinical applications and advance progress toward developing and validating new imaging biomarkers in the field of neuro-oncology.

## Author Contributions

All authors listed have made a substantial, direct, and intellectual contribution to the work and approved it for publication.

## Conflict of Interest

The authors declare that the research was conducted in the absence of any commercial or financial relationships that could be construed as a potential conflict of interest.

## Publisher's Note

All claims expressed in this article are solely those of the authors and do not necessarily represent those of their affiliated organizations, or those of the publisher, the editors and the reviewers. Any product that may be evaluated in this article, or claim that may be made by its manufacturer, is not guaranteed or endorsed by the publisher.

## References

[1] Ostrom QT Patil N Cioffi G Waite K Kruchko C Barnholtz-Sloan JS. CBTRUS statistical report: primary brain and other central nervous system tumors diagnosed in the United States in 2013-2017. Neuro Oncol. (2020) 22:iv1–iv96. 10.1093/neuonc/noaa200 33123732 PMC7596247

[2] Rossignol J Srinageshwar B Dunbar GL. Current therapeutic strategies for glioblastoma. Brain Sci. (2019) 10:15. 10.3390/brainsci10010015 31888004 PMC7016852

[3] Buerki RA Chheda ZS Okada H. Immunotherapy of primary brain tumors: facts and hopes. Clin Cancer Res. (2018) 24:5198–205. 10.1158/1078-0432.CCR-17-2769 29871908 PMC6214775

[4] Wong ET Lok E Swanson KD. Alternating electric fields therapy for malignant gliomas: from bench observation to clinical reality. Prog Neurol Surg. (2018) 32:180–95. 10.1159/000469690 29990984

[5] Chawla S Bukhari S Afridi OM Wang S Yadav SK Akbari H. Metabolic and physiologic MR imaging in distinguishing true progression from pseudo-progression in patients with glioblastoma. NMR Biomed. (2022) 2:e4719. 10.1002/nbm.4719 35233862 PMC9203929

[6] Chawla S Wang S Kim S Sheriff S Lee P Rengan R. Radiation injury to the normal brain measured by 3D-Echo-planar spectroscopic imaging and diffusion tensor imaging: initial experience: assessment of radiation injury to normal brain. J Neuroimaging. (2015) 25:97–104. 10.1111/jon.12070 24279509

[7] Chawla S Shehu V Gupta PK Nath K Poptani H. Physiological imaging methods for evaluating response to immunotherapies in glioblastomas. Int J Mol Sci. (2021) 22:3867. 10.3390/ijms22083867 33918043 PMC8069140

[8] Chawla S Wang S Mohan S Nasrallah M Verma G Brem S. Differentiation of brain infection from necrotic glioblastoma using combined analysis of diffusion and perfusion MRI. J Magn Reson Imaging. (2019) 49:184–194. 10.1002/jmri.26053 29676844

[9] Neal A Moffat BA Stein JM Nanga RPR Desmond P Shinohara RT. Glutamate weighted imaging contrast in gliomas with 7 Tesla magnetic resonance imaging. NeuroImage Clin. (2019) 22:101694. 10.1016/j.nicl.2019.101694 30822716 PMC6396013

[10] Chaddad A Kucharczyk MJ Daniel P Sabri S Jean-Claude BJ Niazi T. Radiomics in Glioblastoma: current status and challenges facing clinical implementation. Front Oncol. (2019) 9:374. 10.3389/fonc.2019.00374 31165039 PMC6536622

